# A Framework of Single-Session Problem-Solving in Elite Sport: A Longitudinal, Multi-Study Investigation

**DOI:** 10.3389/fpsyg.2020.566721

**Published:** 2020-11-20

**Authors:** Tim Pitt, Owen Thomas, Pete Lindsay, Sheldon Hanton, Mark Bawden

**Affiliations:** ^1^Mindflick, Sheffield, United Kingdom; ^2^School of Sport and Health Sciences, Cardiff Metropolitan University, Cardiff, United Kingdom

**Keywords:** problem-cleaning, language, reframing, solution-focused, single session therapy

## Abstract

In this 6-year, multi-study paper we summarize a new and effective framework of single-session problem-solving developed in an elite sport context at a world leading national institute of sport science and medicine (English Institute of Sport: EIS). In Study 1, we used ethnography (3.5 years) to observe how single-session problem-solving methods were being considered, explored, introduced and developed within the EIS. In Study 2, we used case-study methods split into two parts. A multiple case-study design (10 cases) was employed in Part one to evaluate how the approach was refined into an effective framework of practice. An individual case-study is then illustrated to detail the framework *in-action*. Collectively, findings realized a framework of single-session problem-solving for use both inside and outside of elite sport that focused on ways to reframe clients’ problems into more ‘solvable’ descriptions. Guidance for psychologists wishing to integrate these problem-solving techniques into their practice are offered.

## Introduction

Sport psychologists working in elite sport operate in fast-paced multi-faceted operational environments that place many demands on their practices (see Fletcher and Wagstaff, 2009; Wagstaff, 2017). In response, practitioners have broadened the philosophies and therapeutic approaches that underpin their work (Fletcher and Wagstaff, 2009; Friesen and Orlick, 2010; Sly et al., 2020). The traditional focus of developing athletes’ cognitive abilities and psychological skills has been noted as not always meeting the needs of client(s) groups nor the discipline at large (see Cruickshank and Collins, 2013; McDougall et al., 2015). The demand to provide efficient and effective interventions both inside and outside the competition arena at an individual, intra-individual, inter-individual and organizational level is an emerging service requirement for psychologists’ working in elite sport (e.g., Portenga et al., 2012; Wagstaff, 2019). Although some guidance for brief interventions has been offered (e.g., Giges and Petitpas, 2000), these have focused on short and informal interactions with athletes. To date, there are no theoretically driven, rigorous, evidence-based methods for problem-solving within the elite sport environment that enable *fast*, *effective* and *impactful* change (see Pitt et al., 2015b; Wagstaff, 2019). Although such methods are not reported in sport psychology, exploration of single-session approaches in other therapeutic domains does exist and has seen an increase in research and practice activity during the last twenty or so years (see Hymmen et al., 2013). Such work may help guide similar methods within sport.

Following Talmon’s (1990) seminal text, *Single-Session Therapy* a number of other single-session therapy reviews have been conducted as the popularity of the approach grew (e.g., Bloom, 2001; Hurn, 2005; Cameron, 2007; Campbell, 2012; Hymmen et al., 2013). Building on these works, Pitt et al. (2015b) reviewed the history of single-session therapy, summarized the characteristics and effectiveness of single-session approaches, and discussed the potential relevance, applicability and future research directions for exploring single-session therapy within a sporting context. A detailed overview of these concepts and issues is beyond the scope of our paper. However, summary of the key messages from across the reviews cited above indicated: a growing body of single-session therapy research exists within psychotherapeutic and other support settings (e.g., social work, mental health services) that has illustrated single-session therapy can be an effective model of practice across a range of therapeutic domains; although a broad range of therapeutic and guiding models of practice were associated with the delivery of single-session therapy (e.g., cognitive-behavioral models, strategic therapy models, narrative therapy) solution-focused techniques were the most frequently adopted; common characteristics of single-session therapy application included pre-session questionnaires, consultancy teams, consultations that were goal directed, and interventions that incorporated the client’s strengths and existing resources. When defining single-session therapy, Hymmen et al. (2013) referred to “*a planned single-session intervention – not to the situation where a client is offered more sessions but chooses to attend just one”* (Hymmen et al., 2013, p. 61, emphasis added). Several potential future research directions were also offered across the reviews cited. For example, and of relevance here, Hymmen et al. (2013) recommended more rigorously designed studies were required to further evidence the effectiveness of single-session therapy across a broadening range of support domains. With specific reference to the potential relevance, applicability and future research directions for exploring single-session therapy within a sporting context, Pitt et al. (2015b) concluded that the methods associated with single-session therapy could potentially provide sport psychology practitioners with a well-suited, effective and efficient means to solve problems. They also noted that research that exploring *how* single-session methods could be integrated and developed within an elite sport environment would be a beneficial first step to advancing understanding in the application of such methods within a sport psychology support context.

In response to the issues noted above, the broad aim of our research was to generate an applied framework for single-session problem-solving for use within an elite sport context. This broad aim was reached through the two interrelated studies presented herein that took place in a high performance sport context at a National Sporting Institute that supports Olympic and Paralympic athletes. Collectively, we overview a 6-year-long period of research that addressed three specific research questions. In study one, our purpose was to understand how single-session problem-solving approaches were being considered, explored, introduced and developed within an elite sport setting. The purpose of study two was twofold. First, we examined how the practice of single-session methods was refined into a coherent, consistent and effective framework of practice. And, second, we aimed to demonstrate how the different techniques involved in the application of single-session problem-solving can be effectively used alongside each other during a single-session. The full data collection period spanned two Olympiads and reflected a significant and prolonged period of immersion within elite sport. The studies featured in this paper were in receipt of ethical approval from the first authors academic institution (reference 13/02/04R).

## Study 1: Single-Session Problem-Solving in Elite Sport - An Ethnography

### Method

#### Theoretical Positioning and Ethnographic Inquiry

Ethnographies remain novel within sport psychology and therapeutic settings (see Krane and Baird, 2005; Suzuki et al., 2005). An ethnographic process suited the specific research question for Study 1 as it enabled detail about a specific group (i.e., a team of sport psychology practitioners), practice (i.e., the consideration, and early exploration and application of single-session problem-solving methods) and culture (i.e., support in an elite sport organization) to be collected with the ethnographic product allowing understanding of the events, behaviors and meaning within these groups, practices and cultures (Hammersley and Atkinson, 2007). Psychosociologists have promoted ethnography’s strengths as a method to gain deep understanding of work-place dynamics and organizational and operational processes from an insider’s viewpoint (see Smith, 2001). Specific to the field of sport psychology, Krane and Baird (2005) noted that ethnography, especially in applied settings, could be used as a way to understand, enhance, and develop new ways of practicing. The specific criteria that shaped our ethnographic approach were guided by the limited ethnographic works in sport psychology related to organizational psychology (e.g., Wagstaff et al., 2012), the discipline in general (e.g., Holt and Sparkes, 2001; Poczwardowski et al., 2002; Gilbert and Trudel, 2004; Devaney et al., 2017), and those from broader psychosocial literatures (e.g., Foley, 2002; Hammersley and Atkinson, 2007; Wolcott, 2008; Case et al., 2014).

The philosophical approach underpinning our research centered on a pragmatic-critical realism perspective (Fishman, 1999; Johnson and Duberley, 2000). Although critical realism alone is an accepted philosophical school of thought, the combining of a subjectivist epistemological stance (i.e., knowledge is subjectively created through social interactions and language) with that of ontological realism (i.e., an external reality exists), has led some to suggest solely adopting a critical-realism stance can lead the researcher to fall into the trap of trying to explain an objective reality by privileging their own subjective thoughts on what that reality is (de Shazer, 1994; Johnson and Duberley, 2000). Therefore, to avoid assuming privileged knowledge, pragmatic-critical realists adopt the idea of ‘practical adequacy’ and align with the premise that the best explanation is the one that generates expectations about the world which can be best used to realize specific goals (Fishman, 1999; Johnson and Duberley, 2000; Heeks et al., 2019). In the context of our study, where our overall aim was to generate an applied framework for single-session problem-solving for use within an elite sport context, knowledge from our work should therefore be evaluated in the context of how successfully it may guide action toward the realization of this particular objective (Johnson and Duberley, 2000). By combining these principles of pragmatism with the epistemological and ontological beliefs of critical realism, this allowed different experiences (i.e., data) to be used to develop a framework of practice, interpreted in terms of how usable they were toward this goal.

#### Context and Participants

The research was conducted at the English Institute of Sport (EIS), a leading supplier of sport science and sport medicine services to Olympic and Paralympic sport in the United Kingdom (The English Institute of Sport, 2019). At the time of study, the EIS provided services to over 40 Olympic and Paralympic sports, and had a workforce of over 350 management, operational and sport science/medicine staff which included a team of approximately 20 sport psychologists. The ethnography spanned two Olympiads, continued for 42 months and reflected a significant and prolonged period of immersion within elite sport. The ethnography was primarily centered at one of the EIS’s five leading sites that housed most of the EIS sport psychologists. Data collection sometimes occurred at three of the remaining regional EIS sites. The primary participants were the team of sport psychologists who delivered support through the EIS’s network. Such participants were provided with a verbal and written briefing about the study rationale, the methods involved and the potential use of data at the outset of the project (or at such a time when they came into contact with the work). All individuals who feature in the ethnography agreed to participate and provided written informed consent for data collection.

#### The Primary Researcher (Ethnographer) and the Ethnographic Process

I, the first author, was the ethnographer. I was a central vehicle in the research, with my role in the field and the analytical and interpretative processes that followed critical to the construction of the findings (see Douglas, 2002; Foley, 2002; Case et al., 2014; Devaney et al., 2017). As my time in the field progressed, I shifted from an ‘outsider’ to ‘insider’ (e.g., taking applied roles within the EIS aligning with a practitioner-researcher status; Devaney et al., 2017). Ethnographers have described the balance researchers must strike across these multiple, and at times conflicting agendas as the ‘contradictory synthesis’ of ethnographers’ insider-outsider role (see Case et al., 2014). To navigate this process, I maintained regular contact with the research team based externally to the EIS who acted as ‘critical friends’ and helped challenge/support the assumptions I made as the research progressed (Faulkner and Sparkes, 1999). As I became more active within the EIS psychology team, separating my ‘ethnographer’ role from my ‘practitioner’ role became more challenging as I was more regularly called upon to ‘participate’ in the setting being studied without becoming too absorbed to observe and analyze what was happening (Merriam, 1998; Case et al., 2014). My awareness of these issues was critical throughout the ethnographic *process*, and was assisted by the use of three different journals associated with the reflexive approach adopted throughout (see Ethnographic Techniques). Finally, and in line with the representation approach used in other domain specific ethnographies, the use of “I,” “me,” or “my” refers to the first author throughout the remainder of the ethnography, whereas “we,” “us” or “our” denotes the research team (e.g., Wagstaff et al., 2012; Devaney et al., 2017).

#### Ethnographic Techniques

In line with other ethnographies, multiple techniques were used to collect data for this ethnography; these were: observations; field notes; formal and informal interviews and the reflexive and reflective journals kept by the first author (see Krane and Baird, 2005; Hammersley and Atkinson, 2007; Wagstaff et al., 2012; Devaney et al., 2017).

##### Observations

Informal conversations and observations formed the backbone of our ethnographic process (Wagstaff et al., 2012; Devaney et al., 2017). During my immersion in the field I was able to subtly eavesdrop, conduct informal interviews, and engage in dialogue with participants about the single-session problem-solving approach as it was being considered, explored, introduced and developed within the EIS. As the ethnography progressed, the EIS teams early attempts to undertake SS problem-solving sessions became a central focus for observations. The consultancy team setup of these sessions enabled me to take an ‘observer-participant’ role in the observation team (Brewer, 2000). Here, I was able to observe the activities and interactions of the team, the primary practitioner, and the client, whilst simultaneously maintaining transparency (Angrosino and de Perez, 2000).

##### Field notes and reflexive and reflective diaries

Extensive field notes were recorded to account for observations and included information about participants’ actions, comments, conversations, and events (Brewer, 2000). These notes remained descriptive in nature (Krane and Baird, 2005), and were kept separate from the other reflexive and reflective journals I kept. I chose to separate my reflexive notes (i.e., how I was interpreting and making sense of my observations) from my reflective notes (i.e., how my experiences were shaping my thinking as a practitioner) to help me to separate my researcher and practitioner roles. This separation encouraged me to maintain analytical distance and to critically examine my own assumptions with regard to my observations and their meaning (see Holt and Sparkes, 2001; Devaney et al., 2017).

##### Formal interviews

Formal interviews were completed with ten sport psychology practitioners. These 10 represented the group of practitioners who had been in the consultancy team in at least two (or more) of the EIS teams’ early attempts to undertake single-session problem-solving sessions. These individuals were interviewed to gain insight into their perceptions of single-session problem-solving approaches as the team were undertaking early explorations and introductions of the methods. These interviews were conducted toward the end of the ethnographic immersion, lasted for approximately 1 h each and were semi-structured in nature. An interview guide was used to provide a framework for the discussion whilst at the same time allowing the freedom to pursue topics of interest that transpired during the interview. Interview questions were developed from the analytical themes that we had been interpreted as the ethnography progressed that related to how the single-session problem-solving approach had/was being considered, explored, introduced and developed during immersion (cf. Holt and Sparkes, 2001).

#### Analysis, Quality Criteria and the Ethnographic Product

Wolcott’s (2008) approach of *description* (what is going on here?), *analysis* (how does it work?), and *interpretation* (what does it mean?) was used to synthesize the data into the ethnographic product. Analysis was an ongoing reflexive process using this approach. As the study progressed, collected data were continually analyzed, interpreted and reflected upon. This involved me immersing myself in the data to identify, interpret and understand (through reflection) themes from the observations (Holt and Sparkes, 2001). Analytical reflections and notes were also made in my reflexive diary as I made preliminary connections between the data, which in turn informed the progressive focusing of the field work (Holt and Sparkes, 2001). The quality and credibility criteria we used to help authenticate the work were guided by Krane and Baird’s (2005) suggestions. Specifically, first, the immersive period in the field was prolonged allowing detailed observation of behaviors and interactions and the development of rapport (cf. Hammersley and Atkinson, 2007; Wagstaff et al., 2012). Second, the use of various data collection techniques provided multiple insights on the themes, connections, and perspectives we interpreted from within the data (Holt et al., 2013). Third, the research team, all active and accredited researcher-practitioners acted as critical friends (Faulkner and Sparkes, 1999), asking questions about observations and interpretations as the field work of the first author progressed. Additionally, and in line with other ethnographies, I had ongoing interaction with members of the EIS sport psychology team regarding the key themes that were interpreted from the observations as the ethnographic process was undertaken. These stakeholders were a sounding board for our interpretations, they acted as a further layer of critical friends asking us thought-provoking questions about our observations, reflections, and interpretations (Wagstaff et al., 2012; Holt et al., 2013). Through the concurrent processes of engaging in data collection, reflection and analysis, we achieved a suitable level of data interpretation in order to capture the main themes associated with the practice of the single-session problem-solving. The ethnographic product is reported in the first person, through the participants’ own voices using rich in-depth quotations with color pseudonyms used to help provide anonymity. These findings are supplemented by first person researcher(s) interpretations that provide a reflective account of how single-session problem-solving methods were being considered, explored, introduced and developed within the EIS.

### Findings

The ethnographic product we present here summarizes the single-session problem-solving methods as they were initially conceived at the EIS. The method is first contextualized, and an overview of the problem-solving approach that was interpreted as being developed during the ethnography (‘problem-cleaning’) follows. Subsequent sections outline the problem-cleaning techniques interpreted as central to the EIS’s early iterations of single-session problem-solving.

#### Single-Session Problem-Solving

Before entering the field, single-session problem-solving had been discussed within the EIS psychology team for around 12 months but lacked systematic examination or practice. In the early phases of immersion, I met with Blue, a senior psychologist to contextualize how single-session problem-solving was being considered by those within the institute. Blue outlined how the initial interest had started:

*“A few years ago, I emailed some of the team*… *I asked them to consider when they’ve been with a client and solved something they’ve been struggling with for a long time. Imagine if you knew that no matter what problem a client arrived with, you could solve it in one session. If that was possible, what would we have to alter about our thinking of change, problems, people, reality and behavior?”*

This exchange was the catalyst for the senior EIS psychology team to read and immerse themselves around brief, strategic, and solution-focused therapeutic approaches, systems thinking and cybernetics, and the philosophical works of Wittgenstein (1953). They also spent time training with clinical psychologists who had expertise in these areas.

Over coffee, Red, the head of the EIS psychology team recalled how when exploring these different areas “…*a new world opened up. How you look at human behavior changes, how you look at language, at cause and effect, and creating change changes, you realize change can happen in multiple ways.*” My analysis and interpretation of other conversations indicated how the psychologists were aligning their thinking toward the development of a method for resolving problems that could be used across the team. Yellow told me a few months into my immersion:

“We explored the history of brief approaches and the philosophy behind them, thinking we could develop something that might help solve some of the long-standing problems we faced on the run into London [2012 Olympic Games]”

Around 6 months into my fieldwork, opportunities to observe early explorations of some single-session problem-solving techniques appeared. Client(s) would contact the EIS psychologists about problems they had been stuck with for an extended period of time (this ‘stuckness’ was interpreted as an important criterion for *when* single-session problem-solving became appropriate). As with many brief therapeutic approaches, a consultancy team approach was used during these sessions. Initially, this involved a primary practitioner and client in the one room, and (with informed client consent) a team of practitioners observing from another room or situated behind the client. Over time, the observation team’s role evolved to mapping the session as it progressed, capturing resources and moments of insight in real time. Consultancy breaks were taken allowing the primary practitioner to communicate with the observation team, agree on the direction of future questions, and/or gain additional layers of insight about the client’s problem.

When analyzing observations and reflections of an early single-session problem-solving session, Red used a phrase that I interpreted as key, when noting to the client that, “…*we’re realizing that, if you’ve been stuck with a problem for a long time, you’re more than likely trying to solve the wrong problem.”* During these early single-session problem-solving sessions, the client’s description of their problem would frequently change, a reframing process that began to be referred to as “*cleaning*” the problem. This notion of ‘cleaning’ a problem stood out to me as I further reflected on a conversation with Purple, who noted:

“…*there has to be an acknowledgment at the end of the session that the problem description they came in with, might not be the problem they leave to solve. That’s a real shift for the individual(s). They come with a mindset that the problem is ‘X,’ I have tried ‘Y’ to solve it, and even though it hasn’t worked they will still hold the belief that the problem is ‘X.’ Success seems to come when clients leave and their perception of the problem is not rooted to ‘X”’*

My analysis and interpretations suggested this ‘shift’ required the practitioner(s) to challenge the client’s assumptions, generalizations, and description of their problem(s), until it was described in a *“solvable”* frame. Over lunch 1 day, Blue and I spoke about how problems were seemingly being re-labeled and changed (perceptions of) during a session. “*It’s not like they’re different problems as such, they’re different descriptions of the same problem,”* he explained. I reflected that the questions and techniques associated with this ‘cleaning’ process enabled the reframing to occur in a single-session, and seemed central to solving problems quickly.

#### Problem-Cleaning

The primary focus of a single-session problem-solving session was to ‘clean’ the client’s initial problem. This ‘cleaning’ process, often taking several hours, involved the primary practitioner using a series of questioning and reframing techniques that challenged the client’s current perception and description of their problem. These techniques acted as mechanisms for the practitioner and client to re-negotiate the description, or “*problem label*” as the team would term it, until the team and client agreed it was possible to design an applicable intervention. As the ethnography continued into years two and three, several techniques became central to this co-creation of a ‘solvable’ problem. Sessions did not always follow a linear pattern, where techniques were used one after another. Instead, I observed that the primary practitioner would often re-visit certain questions or techniques throughout a session, often after the problem frame was re-negotiated (i.e., re-described). Blue explained this process in an interview:

“*Because of the iterative nature of this problem-solving approach, each time you re-label and re-frame it [the problem], we’re dealing with a new perspective of the problem. So, we can explore previous lines of questioning based on this new perspective [of the problem]”*

This relabeling cycle continued until the problem was described within a ‘solvable’ frame which was realized when the problem was described in simple, behavioral terms, and the client appeared certain that if this one thing happened, their problem could be solved. Specifically, the problem would be described within the frame of “…*the one thing that is not currently happening, that if it did happen, would mean the problem would no longer exist, is*….*”* Hence, the labeling, negotiating, and re-labeling of the problem were interpreted as the critical aspects of the problem-cleaning process. For example, in one session with a performance director, the problem evolved from initially being described as a “*dysfunctional team*” issue to a final description of, “…[*the one thing that is not currently happening is] Andrew [pseudonym] is not aligned to performance objectives when challenged.*” The problem was re-labeled several times during the session as: *“a leadership issue”; “a planning problem”; “a lack of joined up communication”;* and *“an absence of benchmarking.”* My analysis and interpretations led me to find that the re-labeling of problems enabled clients’ perception of their situation to be reframed, giving them greater agency, and helped them understand the assumptions they were making about their situation. Blue, explained why this process was critical:

“… it’s like you’re climbing a mountain and when progress is made you consolidate that gain by ‘pegging the rock face.’ And you think, well if we fall, we’re not falling further than that peg. So, particularly when there’s been some sort of insight, you try to re-label it, because at that point they’re open to seeing the world differently, and the re-labeling acts as a placeholder in the conversation that says, ‘we’re seeing the world differently now,’ so just peg that.”

The observation, analysis and interpretation of the ethnographic data led us to suggest a number of distinct questioning techniques were used during the problem-cleaning phase; each are detailed below.

#### Problem-Cleaning: Initial Checks

As the ethnography continued, I observed a pattern whereby each session began with a series of initial checks that, as Pink commented, were used to “…*get the client to empty their pockets, so everything’s on the table.”* These checks framed the initial problem, and were interpreted as increasing a client’s motivation to think differently about their situation. The first check asked “…*how specifically is this a problem for you?”* This question seemed impactful when used in context, as it fostered agency and ownership by the client and ensured they felt responsibility or accountability over problem resolution (i.e., it helped them to describe how they experienced their problem, rather than describe it from a broader, more general perspective). The primary practitioner would also ask, “…*if you didn’t do anything, and purposefully decided to stop trying to resolve this problem, what would happen?”* This question appeared to be used to enhance motivation and to understand if the client was actively seeking change.

The “*fire alarm question*” was another check used during this initial phase*;* here, the client was asked, “…*if a fire alarm went off now, the session finished and we had to leave straight away, what is the one thing you would try to solve this problem?”* This question helped uncover whether clients sense they knew what they needed to do to solve their problem, but something was preventing them from doing so (which was often revealed by asking a follow-up question of “…*so why haven’t you done this?”*). Finally, during these initial checks, the client would be asked the “*door handle question,”* to establish a clear goal for the session (i.e., creating a goal directed session). As Red asked one client, *“*…*when you turn the door handle and leave at the end of today, what do you need to leave with for this to have been a completely worthwhile process?”*

#### Problem-Cleaning: Exploring Previous Solutions

Exploration of clients’ previous attempts to solve their problem was interpreted as another feature of the problem-cleaning process. Clients would be asked to exhaust every previous solution they had tried (e.g., *“I’ve spoken to them about it,” “We’ve run education workshops,” “We’ve taken disciplinary action”).* These failed solutions were recorded on sticky notes as they were described. The client would then group the interventions that had: made the problem worse; made it somewhat better; or, had no impact at all. Here, a key feature involved the client and practitioner identifying connections between these previous failed solutions. As Red noted, “…*the relatedness of these things [failed solutions], what they’ve got in common, starts to unveil the assumptions clients have made about their problem].”* For example, solutions may have all involved educating or motivating an individual or group, they may have all been delivered by the same person, in the same environment or same context. During an interview, Blue noted how such assumptions prevented problems from being solved, *“*…*you become artificially stuck by how you define the problem, your role, and therefore by what solutions you’re ‘allowed’ to try*… *unpacking those connections reveals how client(s) are stuck by their own assumptions.”* The identification of these assumptions and finding connections between previous solutions in each of the groups (i.e., what had made the problem worse; made it somewhat better; or, had no impact at all) often changed clients’ perceptions of their problem and helped them re-label it. Summarizing this reframing process, Blue suggested, *“*…*when you challenge or identify those assumptions, it opens up a new world of how to describe the problem more effectively, and what other types of solution we might consider.”*

#### Problem-Cleaning: Describing a Preferred Future

Pioneered by brief solution-focused therapists, the ‘miracle question’ is a psychotherapeutic technique whereby clients describe a preferred future, a time when their problem no longer existed (de Shazer, 1985). Influenced by the miracle question, the EIS psychologists used preferred future questions during their early explorations of single-session problem-solving. These helped clients think about a context where their problem had disappeared, identified the goal for resolving the problem, and prevented clients making causal explanations of their problem. Similar to the miracle question, this helped clients consider an imaginary context where they left their sport for a period of time and, on return, without knowing how or why it had happened, their problem had disappeared. This was achieved by asking the client to imagine they had gone on holiday for an extended period of time, for example:

“Imagine you go on holiday for 6 months, and when you’re away you have no contact with the sport. But, when you return, the [problem] is completely gone, it no longer exists. How will you know it no longer exists? What would be the first small signs that this [problem] has gone?

With a description of the preferred future, the primary practitioner often challenged clients to discriminate between what the EIS psychologists termed “*nice to haves*” and “*need to haves.*” ‘Need to haves’ were the most essential elements of the client’s preferred future description (e.g., “*they would make sure to book out the gym when they needed to use it*…*”)*, whereas ‘nice to haves’ were aspects that perhaps included generalizations (e.g., *“they’d always follow the rules, and wouldn’t cause any distraction for the rest of the team”*), or perhaps contained more personal meaning (e.g., “*they’d be more professional, be a good team player, and would respect the rules*…”). This filtering of information acted as another opportunity to challenge the client’s assumptions about their problem. Identifying ‘need to haves’ and ‘nice to haves’ challenged whether a client’s expectations of their problem-free future were realistic or not; and, often led to a different description of the problem being negotiated.

#### Problem-Cleaning: Searching for Exceptions

Searching for “*exceptions*” by exploring times or contexts where a clients’ problem was not present, or was not as prevalent as expected was also used to ‘clean’ a problem. This technique had its origins in solution-focused models of practice (e.g., de Shazer, 1985; O’Hanlon and Weiner-Davis, 2003). The primary practitioner would ask questions such as, “…*has there ever been a time when you haven’t had this [problem]?”* or “…*can you think of a time when [problem] hasn’t been as bad as you’d have expected it to be?”* or, “…*can you think of a time when [an aspect of the client’s preferred future description] has occurred?”* This helped clients consider times when aspects of their preferred future had occurred, and when identified, enabled the primary practitioner to ask the client, *“*…*what’s different about the times when this is less of a problem?”* Revealing exceptions assisted the primary practitioner and client to negotiate the current problem label by using them as reference points to compare against the current frame of the problem. Searching for exceptions also revealed resources that could be used to design interventions. I overheard Blue explaining to another psychologist, “…*exceptions help people identify the resources and uniqueness of situations where the problem disappears or is not quite there.”* After revealing an exception to their problem, the client was encouraged to consider, *“…how could you make that happen more often?”*

#### Problem-Cleaning: Revealing Constraints

Analysis of ongoing observations and field notes demonstrated that practitioners placed value on avoiding causal explanations when describing a problem. Instead, the team referred to “*constraints*” as a means of conceptualizing behavior and behavior change. Here, clients were required to consider what factors were preventing change from occurring, rather than what would cause the change to happen. Blue, explained the relevance of this thinking:

“…*describing the problem in a way that says why it isn’t any other way gives you options for change. If you say the situation is the way it is because of all these things keeping it there, it makes you think of the problem situation being just as unique as an exception. But people normally come with the view that the problem situation is the default. And, we are trying to move it from that default to something special. And that’s not true. The problem situation is just as unique as any, and by getting them to think what has to be there to prevent it from being anything else, you open up a world of opportunities which you delete if you assume the problem situation is the default”*

This way of thinking was interpreted to link heavily to the purpose of exceptions. Throughout all my observations, the team would encourage clients to become curious about exceptions, especially the contextual differences between conditions of an exception and those when the problem was present (e.g., the people present, location, time, rules, actions, interactions). My analysis indicated that by viewing the current problem as being maintained by constraints, the exception situations provided insight into either: (a) the constraints that have prevented change (i.e., contextual details not present during an exception); or (b), any constraints that were present when the problem did not occur (i.e., contextual details that were only present during the exception). Red reminded me of this when in the office 1 day:

“…*by thinking, what’s the thing that’s stopping it from being the exception, it forces an effective description of the problem. Rather than, if you assume causes, you can get a really sloppy description of the current state, full of assumptions, deletions, and distortions*…*if you force them to describe it through the lens of constraints you get a much more effective description with multiple points of intervention available”*

Interpretation of observations led me to note that the primary practitioner would contextualize this way of thinking by asking clients to consider a fictitious situation whereby they had to *guarantee* their problem would exist to further help reveal constraints. This question, was almost the reverse of the preferred future questions and was asked as, “…*if you had to re-create this problem in an alternative universe, from scratch, what would have to be present?*” Responses were often compared to any exceptions that the client had identified. This clarified if there were any constraints (i.e., contextual details) that were not present during the exception. If missing, the reasons ‘why’ they were not present in the exception situation were explored.

#### Problem-Cleaning: Video Descriptions

The team referred to “*video descriptions*” as an important way to challenge a client’s assumptions about their problem. Video descriptions were concrete, behavioral descriptions of a situation detached of meaning, abstractions, and generalizations (O’Hanlon and Wilk, 1987). This often focused on the language clients used. For example, when describing an aspect of their problem as “…*one person not trusting another member of their team,”* the client was asked, *“*…*how specifically do you know that he doesn’t trust them? What tells you this?”* The client’s description of this situation then changed to *“*…*[this person] does not allow [another person] to access the lab without other members of staff being present.*” When asked about this, Blue explained the relevance of these follow up questions:

*“*…*if the problem is that they don’t trust them, I don’t know how to solve that. Whereas, someone walking in and speaking to another person, or allowing access or not to a lab, well we are better positioned to help there. We have no idea what ‘trust’ looks like, we could assume we do, but then we add more noise, rather than clarity to their thinking.”*

#### Gathering and Utilizing Resources

Brown explained during a lunchtime conversation that “*resources*” were described as, *“*…*anything unique to the current situation or problem.”* As the ethnography progressed, my observations led me to interpret that the observation team was central to recording any resources mentioned by the client. Resources were considered useful when designing a co-created intervention. More specifically, resources utilized in interventions included individual skills and interests, metaphors used by the client, and/or specific contexts or environments that were associated with previous positive experiences (often recorded when describing exceptions). Once a ‘solvable’ frame to the problem was reached, the client and primary practitioner joined the observation team and explored any resources that could help refine the details of the intervention. These resources shaped the context of the intervention to ensure it was both memorable and impactful. Red summarized this approach (i.e., using the resources the client brings to the session):

*“*…*Let’s say the one thing that is yet to happen is a crucial conversation with somebody else. What we’re trying to do with resources is consider where that conversation will be, when or how to make the message land, and what’s going to make people listen so the message sticks.”*

### Study 1: Summary

In Study 1, we used ethnography to observe how the EIS psychologists considered, explored, introduced and developed single-session problem-solving within the specific context of sport psychology support within the United Kingdom. Our observations, analysis and reflections led us to interpret that the approach suited long-standing problems that clients’ felt ‘stuck’ with having attempted several failed solutions to try and resolve the issue. We found that multiple ideas and techniques were introduced and developed when adopting a single-session problem-solving approach. These centered on several stages of ‘problem-cleaning’ that elicited a more ‘solvable description’ of the client’s problem. Intervention design was supplemented with resources gained through this process. We concluded that several of the problem-cleaning techniques used by the EIS team were influenced by, and based upon other brief and solution-focused therapeutic approaches; perhaps most notably, those of Bateson (1972, 1979), de Shazer (1985, 1988) and Erickson (e.g., Wittgenstein, 1953; Watzlawick et al., 1974; O’Hanlon and Wilk, 1987; Zeig, 2014). However, the EIS psychologists’ integration of these techniques into one single-session problem-solving approach represented an original use of these ideas. Using a consultancy team model of practice was also a feature of the approach, something often associated with some single-session therapies (e.g., Fry, 2012; Slive and Bobele, 2012) and brief therapy models (e.g., Watzlawick et al., 1974).

We reasoned that ‘Exploring previous solutions’ was central to problem-cleaning. The EIS teams use of this approach extended the therapeutic work of Watzlawick et al. (1974) and Weakland et al. (1974) that primarily focused on a client’s previously attempted solutions. Watzlawick et al. developed paradoxical interventions by exploring the similarities within previous failed solutions and intervening by doing the opposite of these higher-order connections. The EIS psychologists used the connections between a client’s previous solutions in a different way to reveal assumptions and subsequently reframe a problem. Similarly, the ‘describing a preferred future’ technique observed as being used by the EIS team was developed from the therapeutic work of de Shazer (1985) and de Shazer et al. (2007). The ‘miracle question’ was central to brief solution-focused therapy; we interpreted that the EIS team extended this by using questions that considered essential ‘need to have’ features of a preferred future. The EIS psychologists’ ‘search for exceptions,’ that was used to reveal times or contexts where the problem was no longer present or had not been as bad as expected, was suggested following our analysis to also be influenced by brief solution therapy (e.g., O’Hanlon and Wilk, 1987; de Shazer, 1988).

Our observations and analysis indicated the EIS psychologist’s problem-cleaning technique of ‘revealing constraints’ shared similarities to the philosophical and theoretical work of Bateson (1972, 1979). Specifically, as an alternative to causal explanations of behavior embedded in linear scientific thinking (e.g., snooker ball A moved in a certain direction because snooker ball B hit it at a certain angle), Bateson outlined a cybernetic negative explanation to help understand complex problems (e.g., what prevented snooker ball A moving in any other direction that the one it took). Bateson’s perspective suggested behavior could be considered via alternative outcomes that were conceivable, followed by an approach that questioned what was preventing these alternatives from being achieved. We interpreted that the EIS psychologists use of ‘constraints’ extended Bateson’s thinking to an applied technique to aid problem-solving, by asking clients what was preventing their preferred future or exceptions from being a consistent reality. A particularly novel finding was the use of questioning techniques that helped consider how a client(s) problem would be guaranteed to exist in another context. This allowed clients to reflect on what was preventing their problem from being resolved and gain agency over these influences.

Another of the problem-cleaning techniques we observed being used during single-session problem-solving were ‘video descriptions.’ This technique aligned with the work of O’Hanlon and Wilk (1987) who described such descriptions as an essential way of *“*…*sifting facts from meanings*” (p. 17). In this context we interpreted that ‘facts’ were observable events that could be verified (i.e., observable on a video screen, without interpretation); whereas, ‘meanings’ related to the evaluations and interpretations that clients made about their problem (O’Hanlon and Wilk, 1987). From this perspective, we began to understand how the clients’ problems were being re-framed; specifically, due to the different ‘meanings,’ interpretations and ways of *describing* ‘facts’ or observable events. We found that practitioners used ‘video description’ questions to help clients’ distinguish facts (e.g., allowing another person to access to a lab) from meanings (e.g., a person not trusting someone) in order to help re-describe problems in a more solvable frame (cf. O’Hanlon and Wilk, 1987).

In summary, within our ethnography, we observed that learning, and visible attempts to engage in single-session problem-solving were taking place within the EIS. Our interpretations led us to suggest this approach used resources from a range of brief and solution-focused therapies (e.g., Watzlawick et al., 1974; de Shazer, 1985; O’Hanlon and Wilk, 1987; Zeig, 2014) but also applied some new and novel techniques. However, at this stage of development, a clear framework to underpin the EIS psychologists’ practice of single-session therapy was lacking, and testing the effectiveness of the approach was not undertaken with rigor. Thus, the next stage of the research moved toward attempting to systematically refine a clear framework of practice and demonstrate the effectiveness of the single-session approach.

## Study 2: Single-Session Problem-Solving in Elite Sport – Developing an Effective Framework of Practice

### Introduction and Methodological Positioning

Building on the findings of Study 1, and on calls within the literature to design studies that more rigorously evidence the effectiveness of single-session therapy across a broadening range of support domains (e.g., Hymmen et al., 2013), Study 2 was a two-part case-study that aimed to: first, evaluate how the single-session problem-solving approach was refined into a coherent, consistent and effective framework of practice; and, second demonstrate how the different techniques involved in the application of single-session problem-solving could be effectively used alongside each other *during* a single-session. Case-study methods were deemed an applicable empirical method to meet these aims as they enable contemporary phenomena to be investigated within a real-world context and best suit situations where the main research question(s) involve a ‘how’ question (Yin, 2018).

### Method

#### Design

Several authors have argued for greater use of case-study research within sport psychology and other therapeutic domains to further the systematic development, application and understanding of applied practice (e.g., Anderson et al., 2002; Barker et al., 2011; McMahon et al., 2012). Despite these calls, some view case-studies with veiled skepticism surrounding their methodological merits, with discipline purists questioning their position on causal attribution (e.g., Verschuren, 2003; Kyburz-Graber, 2004). In short, case-study methods may not meaningfully control for several threats to internal validity and suffer challenges around generalizability due to their inherent ‘case’ based inferences (Kazdin, 1981). Despite such issues, others promote its viability for exploring, describing, and potentially explaining contextually embedded psychological phenomena at either the individual or inter level of analysis (e.g., Stake, 1995; Gone and Alcántara, 2010; Yin, 2018). As such, a case-study approach suited our aims as it allowed detailed examination of a contemporary phenomenon (i.e., single-session problem-solving) *within the context* in which the phenomenon occurred (i.e., embedded within elite sport settings). To further help address some of the criticisms case-studies have faced, advocates of such methods developed strategies to improve the rigor within their work (see Gone and Alcántara, 2010). Davison and Lazarus’ (2007) offered six strategies outlining how case-study methods contribute to psychological knowledge: (a) casting doubt on general theories; (b) providing valuable heuristics for subsequent research and practice; (c) demonstrating novel applications of established principles; (d) affording (in some instances) scientifically valid inferences from single-participant experiments; (e) exploring rare but important phenomena; and, (f) contextualizing, illustrating or applying knowledge in particular contexts. Davison and Lazarus’ (2007) indicated case-study researchers should strive to design their studies with such strategies in mind and outline which feature in their work. For our research, which contributed to knowledge by offering a new framework of single-session problem-solving, and for this study (Study 2), that evaluated how the single-session problem-solving approach was refined into a clear framework of practice, explored how the various techniques used operated alongside each other and demonstrated the effectiveness of the approach, we used five of Davison and Lazarus’ (2007) strategies. Specifically, strategies b through f.

Other prominent case study authors have emphasized the need for researchers to clearly articulate the ‘type’ of case study design adopted (Stake, 1995; Yin, 2018). We used a ‘descriptive’ case study approach as this most suited our aims. Such designs allow cases to be represented as natural phenomena and allow for data to be collected within the context of its use (Yin, 2018). To help address our first aim, we adopted a multiple-case study design (10 cases) to evaluate how the single-session problem-solving approach was refined into a coherent, consistent and effective framework of practice. Here we used an adaptive (not closed) multiple-case study design (across 10 cases) underpinned by Yin’s (2018) recommendations (see Data Analysis). To address our second aim, we adopted a single descriptive case study approach to help demonstrate how the different techniques involved in the application of single-session problem-solving could be effectively used alongside each other during a single-session.

#### Participants

Participant (client and practitioner) details and the context surrounding their presenting problem are noted in Table 1. These 10 cases were selected using opportunity sampling methods and included a range of roles within the EIS workforce (e.g., athlete, coach, senior leaders). The practitioners (primary and team) were all practitioner/chartered psychologists (Health and Care Professions Council, British Psychological Society) and active members of the EIS psychology team. To help provide anonymity, gender neutral pronouns are used and all clients and practitioners are provided with a color pseudonym. All participants (client and practitioner) provided written informed consent to participate.

**TABLE 1:** Summary of cases featured in Study 2.

Client role (case number)	Type of problem (as originally framed by the client)	Pre/post questionnaire	Team setup	Follow-up/social validation	Development and redesign phase
Sport scientist (1)	*“A loud, obnoxious, closed minded athlete, whose attitude to training stinks, and is confrontational when talking about this. And, because they’re not training to their level, is about to waste their Olympic Games”*	No	Face-to-face (plus 3 CRP’s)	Informal phone call	Phase 1
Sport scientist (2)	*“A disruptive and difficult member of staff, who never follows a process, whose lack of care for anyone else and poor communication is impacting on all members of the team”*	No	Skype (plus 4 CRP’s)	Informal phone call	Phase 1
Athlete (3)	*“Lost move syndrome on one particular movement in my routine, that has got worse the more I have tried to sort it out”*	No	Face-to-face (plus 2 CRP’s)	Informal phone call	Phase 1
Head coach (4)	*“An athlete with an unmovable performance block, who has lost the ability to perform a routine despite many attempts to re-train their skills”*	No	EIS iPsych (plus 4 ORP’s)	Informal phone call	Phase 2
Head coach (5)	*“A squad of players lacking any professionalism or willingness to change”*	No	EIS iPsych (plus 3 ORP’s)	Formal interview	Phase 2
Sport scientist (6)	*“A stubborn, inflexible athlete who is overtraining and pushing beyond their limits despite being told this by all members of staff and refuses any form of help from the team”*	No	EIS iPsych (plus 4 ORP’s)	Formal interview	Phase 2
Sport scientist (7)	*“A group of athletes who are very inconsistent with their conditioning attendance, and nothing seems to work to motivate them to change”*	Yes	EIS iPsych (plus 3 ORP’s)	Formal interview	Phase 3
Sport scientist (8)	*“A cultural and professionalism problem regarding weight management across the program which has resulted in increased injury risks and prevented some athletes from competing”*	Yes	EIS iPsych (plus 3 ORP’s)	Formal interview	Phase 3
Senior leader (9)	*“A squad of players lacking unity, team spirit, or bond between any of the players”*	Yes	EIS iPsych (plus 3 ORP’s)	Formal interview	Phase 3
Senior leader (10)	*“A fundamental, organizational wide cultural problem that continually prevents us from successfully implementing our values and establishing new ways of working”*	Yes	EIS iPsych (plus 2 ORP’s)	Formal interview	Phase 3

*CRP’s, N practitioners in the consultation room forming the consultancy team; ORP’s, N practitioners in the observation room forming the consultancy team.*

#### Procedure

The single-sessions all took place within one of the EIS’ leading sites within the United Kingdom. Participants attended the venue on an agreed day and time. Participants were either a (self-directed) referral to an EIS sport psychology practitioner or were put in contact with a lead practitioner via another EIS employee to discuss the potential use of a single-session approach. Prior to all sessions taking place, any potential client held telephone conversations with a lead EIS sport psychologist to discuss the broad single-session approach and the possibly sufficiency of it as a viable option for the client based on their situational needs. The means and complexity of pre-screening evolved as the multiple case studies progressed. Other procedural elements also evolved during the adaptive multiple-case study design used across the 10 cases (see Table 1 and Findings). Data for each case were collected using recognized case-study methods and included: observations; interviews; and, group debriefs with the clients and practitioners at the end of each session (Gone and Alcántara, 2010; Yin, 2018). All single-sessions, interviews and group debriefings were recorded for transcription.

#### Analysis

Data analysis of the transcripts from each single-session, associated interviews and group debriefs followed recommendations for use with both descriptive and multiple case-study designs (see Stake, 1995; Yin, 2018). Specifically, when using a descriptive case study approach, the researcher(s) must begin with a descriptive framework to support the pattern-matching process of identifying the critical aspects of the case (Yin, 2018). In this study, the data were interpreted, supported, and described through the descriptive framework outlined from the ethnography undertaken in Study 1. Each case exited as a ‘whole’ study where within-case analysis was used to draw conclusions from the observations, interviews and debriefings about the refinement and practice of the various single-session problem-solving techniques. As the multiple-case studies progressed, cross-case analyses were undertaken with the same goal. This adaptive cross-case approach included the feedback loop advocated by Yin (2018) to allow ‘redesign’ of the single-session problem-solving method as it developed into a refined framework of practice. The first author completed first stage analysis following each case. Once these critical aspects of the case (or refinement) were realized, a peer debriefing meeting was held with the research team. These meetings were used to discuss each case and the application of single-session problem-solving methods as it was refined into a framework of practice until agreement on the stages of the developing framework were agreed. These changes are displayed in Table 1 and included: client role, type of problem, pre/post questionnaire, team setup, and follow-up/social validation.

### Findings

The findings are presented in two parts in line with the two aims of Study 2. Specifically, first we present the findings from across the multiple-case study element of the study where we aimed evaluate how the single-session problem-solving approach was refined into a coherent, consistent and effective framework of practice. We then summarize a single descriptive case in detail (case 10) to demonstrate how the different techniques involved in the application of single-session problem-solving could be effectively used alongside each other during a single-session.

#### Refinement to a Coherent, Consistent and Effective Framework of Practice

Following the adaptive multiple-case design used (Yin, 2018) to address our first aim, the cases were divided into three development and redesign phases that realized the final framework of practice. In phase one (cases 1–3), clients requested the session due to feeling stuck with a particular problem that they had failed to solve despite attempting multiple solutions. For these cases, practitioners in the observation team were in the same room as the primary practitioner and client. Regular breaks were taken so the observation team could share their thoughts, observations, and reflections with each other and the primary practitioner. Guided by previous team consultancy models (e.g., Selvini and Palazzoli, 1991; Fry, 2012), the observation team recognized they needed to discuss ideas as the session progressed. Hence, during phase two (cases 4–6), the EIS developed the iPsych system to allow ‘real time’ observation and discussion of the session from a separate room (see Pitt et al., 2015a). The client and primary practitioner returned to the observation room once a ‘solvable’ frame to the problem had been reached. This ensured the observation team helped design the intervention and allowed any resources captured by them to be included (Willott et al., 2012). During this phase, clients still requested sessions as they felt ‘stuck’ with their current problem. However, unlike the first phase, all cases were conducted with non-athlete stakeholders. One EIS practitioner noted, “…*we realized this approach is not easy to do when someone is struggling with themselves in a situation, unlike when they come with a problem of ‘I’m struggling with this person, or this group or this context,’ or this problem that’s about the system,’ then it works well.”* This trend continued until the end of the study emphasizing that psychologists working in elite sport support varied client groups and operate at an organizational level (Sly et al., 2020). Finally, during this phase, the follow-up evaluation process was refined into a formalized semi-structured interview that also acted as a social validation mechanism to determine the perceived effectiveness of the intervention (e.g., Kazdin, 2011).

**FIGURE 1:**
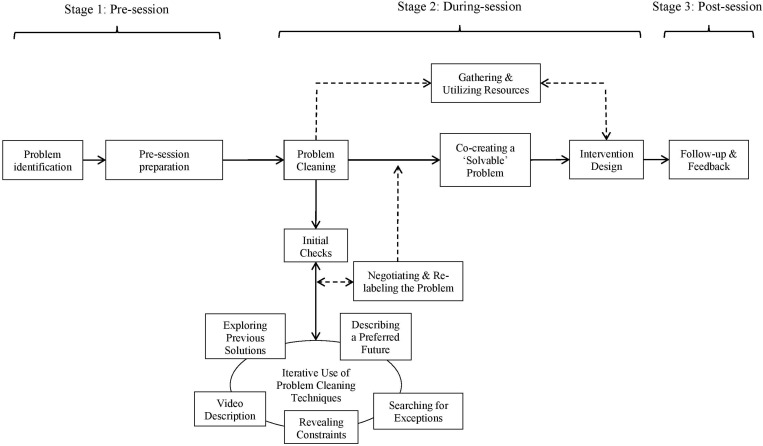
An effective framework of single-session problem solving.

During the third phase of development and redesign (cases 7–10), the EIS practitioners identified the need to obtain more data from clients prior to their arrival. Hence, an eight-item pre-session measure based on the items from the Problem Evaluation Survey (PES; Campbell, 1999) was developed. Open-ended items were added to gain detail on the client’s initial frame of their problem, their previously attempted solutions, and what they wanted to gain from the session. Responses became a suitable point to begin the session, and assisted clients’ recall of the solutions they had previously attempted. Alongside the social validation interview, the adapted PES measure was also used post-intervention to help evaluate effectiveness. The final case (10) was conducted using: the iPsych system to facilitate a consultancy team model; pre- and post-session questionnaires; and, a formal follow-up social validation and is illustrated in Figure 1.

#### A Detailed Single Descriptive Case

Case 10 was selected as the descriptive case used to address the second aim of Study 2 as it represented the EIS’s single-session problem-solving framework as an effective framework of practice. A detailed, descriptive case allowed us to capture the complexity of a real-life consultancy; and, more specifically in response to our second aim, the effective application of the different techniques used from within the framework *in-action* from start to finish (Yin, 2018).

Case 10 lasted over 4 h and was transcribed verbatim (47,377 words). The transcripts and supplementary data (e.g., pre/post session questionnaire, observation teams’ records, follow-up social validation interview transcripts) were analyzed using pattern-matching logic (Zainal, 2007; Yin, 2018) to identify the critical aspects of the case (based on the findings Study 1). An abridged version of critical sections of the case is provided in Table 2. This presents the central techniques of the single-session problem-solving framework and illuminates how they were used alongside each other at this final stage of testing. To enhance the credibility of the findings (Tracy, 2010), two researchers analyzed the data independently, cross-referenced their selections, realizing the narrative in Table 2. Mellick and Fleming (2010) outlined the sensitive nature of narrative reports, and cautioned that a pseudonym alone might not be enough to anonymize data in cases derived from an elite sport context. Therefore, alongside the use of a pseudonym, contextual details relating to the client have been modified to enhance anonymity. The client reviewed and signed off the case report as a credible representation of the data (Stake, 1995; Yin, 2018). The findings are separated here and presented in relation to the main stages illustrated in Figure 1.

**TABLE 2:** Critical techniques and integration of SS problem-solving methods: An abridged summary of the case example of Navy.

Problem-cleaning technique(s)	Practitioner–client interaction	Description
Initial checks	Red: So, our first question is, why are you here today? Navy: Because there’s a lot of pressure, stress, and difficulty about the change of the culture that fall on me, because of the job I do and the responsibility I carry. There’s a significant problem instilling values across our organization. And, sorry, when I talked to you before, I thought it’s a bit selfish of me coming here, it should be other people, and, I should go last! But, I feel as if I owe it to the organization, and maybe to myself. Red: That’s interesting, because often people think they’ve got to send other people, but actually it starts with you. I’m interested though, how is this a problem specifically for you? Navy: Well, I’m in charge of putting the culture and values program together, but nothing seems to land as we have misalignment in the organization. If this was an orchestra, and I could stop the music mid-concert and just enable this change, then that would still be a challenge, but easier. But it’s hard while there is so much going on and people going in different directions… Red: So, we have the culture of the organization moving forward, and then you’ve got an alignment issue. Do you see these things as entwined or separate? Navy: Well, we’ve been trying to instill a new vision and values… and it’s being realized, but we need it. We owe it to our people to give purpose and clarity about where we’re going, how we’re doing, and how they can contribute. Yet, I see this big elephant in the room. I think, in terms of the organization moving forward, most of the leadership is on the bus, but some are not, and I want to make that right. And, those two things are converging, but are in conflict. Red: Can I ask, in terms of the alignment in the leadership team, how do you know there’s a problem there? Navy: Because we don’t have a consistent message feeding through the organization from the leadership team. People on the ground often report to me that they don’t feel listened too. Red: Okay, imagine you decided to do nothing with this problem, you said, “okay, I’m not going to do anything about this, I’m just going to let it play out.” If you stepped back and did nothing, what would happen? Navy: Well, the world’s not going to stop, people aren’t going to die. So, it’s not going to be a tragedy. But, we’ll have let people down if we don’t do something. Red: Who will be let down? Navy: Our employees. It won’t be a tragedy, but we’ll have let people down, and I’ll feel personally responsible if we do nothing. Red: If you could do anything to fix this problem, and you had to do it right now, what would you do? Navy: I’d get the leadership team aligned first, before doing anything with the organization’s values. I’d make sure our leadership team was singing from the same hymn sheet. Red: So, that’s interesting, it seems like you’re saying this is more of a leadership problem than a whole culture problem? Navy: Yeah, I guess it is actually… Red: Okay, so with all that in mind, what do you need to leave with today for this to be a really successful use of your time? Navy: A plan of action and a way forward… so I no longer feel so stuck. I would like to feel less stressed about the whole thing. Red: Okay, so if we’re framing this problem, like, in the title of a Sherlock Holmes book, what would this problem be the case of? Navy: I’d say [long pause]… the case of the lack of leadership alignment.	Red used initial check questions and established the client perceived responsibility for the problem, had a clear goal for the session, and assisted Navy to re-label the problem.
Exploring previous solutions	Red: Can I ask, do you think these solutions have anything in common? Navy: Not really, no I can’t see anything obvious. Red: Okay, is there anything similar you notice about these interventions, anything at all? Navy: [pause]… well they’ve all involved education or up-skilling our staff. Red: Okay, do you notice anything else? Navy: Well actually, they’ve all been aimed at our staff and the leadership team haven’t been involved in the solutions. They’ve all been organization wide. That’s interesting isn’t it… Red: What’s interesting? Navy: Well, we’ve, I’ve seen this as a problem with the culture on the ground, and in doing so I’ve assumed that all the leadership group is on board. I think that clarifies for me that we’ve been looking at culture as a whole, when actually we should be starting with leadership.	After exploring all previous attempted solutions, Red probed the client to look for similarities between them. This revealed assumptions Navy had made about the problem.
Describing a preferred future, video descriptions, revealing constraints	Red: So, we got to the case of the lack of leadership alignment. So, let’s just suppose that you went on holiday for a few months. And when you’re away a miracle occurs and this problem is solved. When you come back to work, how would you know this problem has gone away? What would you see that makes you realize this problem no longer exists? Navy: I would see engagement, clarity, and motivation in others… I’d see people smiling and feeling part of something bigger. Red: And when you say people, are we talking about players, coaches, staff? Navy: I don’t know, as no one has said I’m not being coached right, but I believe it’s the classic of, if your staff are happy, your customers are happy. I think I would know though as it would be the staff talking like this. Right now, I think they’re feeling the brunt of the leadership misalignment, but unlike me they don’t feel responsible for it. They see the consequences, but it’s not their problem. Red: That’s why you’re here. So, let’s go back to our miracle, the miracle’s happened, what else would you notice? We’d see motivated and engaged staff, but what else would you see? Navy: I’d see a few people, not all, because I don’t think everyone is seeing the problem in the organization, but some would be relieved that something had changed. And, if the problem had gone away I think we might see some players come through in the long term. Red: Anything else? Navy: [pause]… I’d see the leadership team providing inspirational direction, showing everyone the direction of travel. Red: What else would you see? Navy: I’d see people knowing their roles, collaborating around a core vision, and just feeling part of something special. Red: Going back for a moment, in terms of engagement and staff motivation, the leadership team providing direction, who is doing what in this miracle future? Navy: Little things, our staff would have clear objectives and know how those aligned with our overall vision, they’d know when each other are taking holidays. Our leadership would have a united message to staff about our aspirations and the direction we want to go, and would be aware of the temperature of the staff on the ground. Red: By temperature, what would you actually see if this was the case? Navy: Well… the leadership team would be aware of issues that the staff faced, and would be working with them to resolve them. Red: So, correct me if I’m wrong, in the miracle future there would be staff on the ground smiling, collaborating together, feeling motivated, engaged, and part of something special, being led by a leadership team who were aware of any issues and working with the staff to resolve them. I know we said miracle, but this is quite idealistic at the moment, a few ‘nice to haves’ as we call them, are there any aspects that are absolutely essential for this problem to no longer exist? Navy: Well, I suppose it would be nice if everyone has smiling faces, and actually, it is just a ‘nice to have’ that everyone is feeling part of something special. That would be a hard aspiration to achieve [laughs]… Red: Okay, let’s look at what we absolutely need for us to know this miracle had occurred and your problem no longer existed? Navy: I think one aspect that we need is to have people coming together and collaborating around a central vision. Getting the most out of each other… Red: Really? You really need collaboration for this issue to be gone? Navy: Well, probably not actually [pause]… the thing we absolutely need would be the leadership group being aware of, and responding to issues on the ground. Red: So, can I just ask, what stops this from happening? Navy: I’m not sure if they’re fully aware of the noise on the ground. Red: That’s interesting, so you’re saying the leadership team isn’t fully aware of the concerns of staff on the ground? Navy: Yes. Red: Does the problem feel like it’s moved again to you? Navy: It does, I think that it’s not so much of a leadership alignment issue [pause]… I’d say it was now the case of the unaware leadership team.	Red helped Navy describe a preferred future. This was supported by video descriptions and questions aimed at revealing constraints. This helped Navy reframe the perception of the problem.
Searching for exceptions and revealing constraints	Red: So, we’ve reached the case of the unaware leadership team. Has there ever been a time when this hasn’t been a problem? Navy: Hmm… I’m not sure there has. They’ve never been aware of the noise coming from the staff that I receive. Red: So, you’re telling me the leadership team has never been aware of any noise from the ground? Navy: No, I don’t think they have, never to the extent that I have. Red: So, they must have been slightly then at some point. Have you ever seen a time when the leadership team has been aware of the noise from the ground? Navy: Actually, ironically [sighs]… about 18 months ago I sat down with the boss and went through the staff survey. That’s when we identified staff engagement and the culture as being something we really needed to work on. This is actually the source of where many of the interventions we mentioned earlier came from. But after that conversation, there was a clear period when members of the leadership team were very aligned in their message to staff and were working with them. And, because of that, staff responded well. Red: And what has stopped that from happening? Navy: I think my work on the cultural side of things. I’ve identified people need to feel connected to a consistent message from above. But I think I’ve seen this as my problem to solve with the culture and values project. But the alignment only really comes from the boss, and when there’s a strong message coming down from above. The leadership team kept to the same message and worked closely with the staff, who themselves definitely felt this. I think I’ve been focusing attention in the wrong place…	Red searched for exceptions. By identifying an exception, Navy gained a critical insight into the problem.
Initial checks and solvable problem	Red: Okay, with that in mind, how specifically is this a problem for you now? Navy: This is a problem because I have never given the boss feedback about the staff’s perception of the leadership team’s performance [pause]… and because I’ve seen this as my problem, up to me to solve, it’s been my project around the culture and values of the staff. Red: So actually, what is your problem then going back to our case of…? Navy: For me, this is the case of the difficult conversation. Red: And, why is that the case? Navy: Well, it’s the case of the difficult conversation because you only really get one go at having tricky conversations with my boss. In fact, the last time I tried to influence the boss about a different area of the organization it didn’t go well. Red: Okay, so I think we’re getting somewhere. Do you think we can say that there is one thing that’s not currently happening, that if it did, it would move this forward and even lead to your problem being solved? Navy: We are! I think the one thing that’s not currently happening is that I’ve not ever had a clear, frank conversation with my boss to share the feedback I consistently get from the staff on the ground about the leadership team and the lack of direction. This is my actual problem. Trying to get my boss to get the leadership team on a strong and consistent message – which I know he will.	Red and Navy negotiated a final and ‘solvable’ frame to the problem by revisiting an initial check question.

##### Stage one: pre-session

The client, Navy, was employed in the senior leadership team of a large United Kingdom sports organization. Navy was responsible for people and culture and contacted the EIS psychology team about being unable to solve a particular problem. After two initial discussions on the telephone, Navy completed the pre-session questionnaire that captured detail on the perceived frequency, severity, interference, worry, coping approaches used, and sense of responsibility for the problem (cf. Campbell, 1999). Before the session started, Navy spent time in the observation room with the team and talked through this background information. Navy had worked for the sport for several years, was considered a high performer, possessed an excellent awareness of culture, leadership, people management, and was familiar with modern approaches to achieving large organizational cultural change. Navy had a genuine drive to make a difference, resolve the problem, and make a tangible difference to the sport for performance benefits and wider societal benefit across the United Kingdom. Navy stated the problem was “*A fundamental cultural problem throughout the whole organization that continually prevents us from successfully implementing our values and establishing new ways of working.*” It manifested in poor performance against KPI’s, an under-utilization of resources, high staff turnover, and political infighting amongst the leadership team. For the past 18-months, the sport had been trying to make cultural changes but little progress had been made and that they were stuck with this issue.

##### Stage two: during session

Following initial discussion with the team, Navy and Red (primary practitioner) moved to the consultancy room and the other EIS psychologists remained in the observation room. The primary practitioner asked the initial check questions and established Navy felt some responsibility for the problem and had a clear goal for the session (see Table 2). This assisted Red and Navy to negotiate and re-label the problem for the first time (to a “*lack of leadership alignment*”). Red used the prefix “*a case of*…” to increase the significance of this moment priming a sense of curiosity in the client. Next, Red and Navy explored, and recorded on sticky notes the solutions Navy had attempted over the last 18 months to resolve the problem. These included: value-based workshops with all staff; creation of ‘cultural champions’; communication training; staff surveys; cultural awareness education; character profiling; and team away days. Red assisted Navy to look for connections between these previous solutions, allowing Navy to recognize how the original “*cultural*” frame of the problem was preventing focus elswhere (e.g., leadership team, see Table 2).

A short break was taken that allowed Red and the observation team to share reflections, before Red returned to the consultancy room to ask Navy to describe a preferred future. Navy was asked to describe how, in a fictitious future where the problem no longer existed, he would know that the problem was resolved. Red used video descriptions to help the client describe and challenge his preferred future (e.g., “…w*hat would you actually see if this was the case?*”). ‘Nice to have’ and ‘need to have’ elements were explored helping Navy reframe the perception of the problem from a “*leadership alignment*” issue to an “*unaware leadership team”* issue. Within this different frame, Red searched for exceptions. When doing so, Navy recognized that approximately 18 months ago there was a time where the leadership team had given a consistent message to staff. By identifying this exception, Navy gained a critical insight into the problem. Demonstrating the iterative nature this problem-solving method, Red re-asked Navy the initial check of how the problem was now an issue for him. This resulted in the problem being re-negotiated to “*the case of a difficult conversation.”* The problem was now considered ‘solvable’ as Navy could described the one thing that was not currently happening, that if it did, might lead to the problem being solved was: “…*a clear conversation with my boss to share the feedback I consistently get from the staff on the ground about the leadership team and the lack of direction.”*

Once the problem was framed in this solvable manner, the problem-cleaning phase was complete and Navy and Red returned to the observation room so the client and entire consultation team could consider potential solutions. The observation team had ‘mapped’ out the session on a large whiteboard whilst the session was underway; this map included a list of potential resources that might be utilized in the intervention. Using the contents of the whiteboard to facilitate the conversation, the team helped Navy consider how, where, and when to have the difficult conversation with the manager, during which they discussed the feedback Navy had received about the leadership team from the staff.

##### Stage three: post session

The scores from the pre- and post- session questionnaire revealed perceived frequency, severity, interference, worry, and sense of responsibility for the problem decreased post-intervention. The social validation interview was conducted with the client 6 weeks post-intervention. Navy indicated being very satisfied with the intervention, stating the session was very useful in helping clarify the *actual* problem that needed to be resolved:

“The most important thing was to take one problem and properly diagnose it. Really understand what my problem actually was - rather than something else interfering with what you’re talking about. So, that time, to really investigate one issue was very beneficial. Often, we don’t spend enough time at that diagnosis phase – so, therefore, the remedy isn’t necessarily properly thought through.”

Navy stated purpose and direction surrounding the problem was evident after the session; and, that the confidence to have the ‘difficult’ conversation with the senior figure had been gained. Navy reported the situation was no longer a problem, the conversation with the boss had taken place in line with how it was designed in the intervention phase of the session. Navy’s boss had listened, and undertaken an appropriate level of action to ensure that a consistent message and approach was being delivered by the leadership team:

“It is no longer a problem. It’s been dealt with, and it’s been dealt with in a better way as a result of me being able to influence someone else, in a different way than I was intending to do. So, yes, the problem has gone away.”

Finally, reflecting on whether Navy would recommend the approach to colleagues, and in what situations it might be fruitful, Navy remarked, “…*when you’ve got lost in a problem, to a point where you can see no way out, and you know that you need to see a way out, because it’s your job to do that, then that is when to consider this approach.*”

### Study 2: Summary

In Study 2, we used case-study methods to demonstrate the systematic refinement and effectiveness of the single-session problem-solving method across 10 multiple cases as it was redesigned into a final ‘framework’ of practice. The final iteration of the single-session problem-solving framework is provided in Figure 1. This framework was separated into three main stages (pre-session, during session, post-session). Stage 1, the pre-session stage, assessed if applying the single-session problem-solving framework would likely suit a client’s presenting problem. Suitable problems were often longstanding, were characterized by clients’ attempting a number of previous (failed) solutions, and involved clients who reported feeling ‘stuck’ with their problem (cf. Talmon, 1990). Clients often communicated a frustration of not being able to resolve the problem and perceived the original frame of their problem as ‘complex.’ Problems were often labeled as multi-agency in nature, involved a number of other people, and crossed both people and organizational issues (cf. Wagstaff, 2019; Sly et al., 2020). The problem identification criteria, and associated indicators for each of these criteria are presented in Table 3. The pre-session stage also included use of an adapted version of the PES (Campbell, 1999).

**TABLE 3:** Problem identification criteria and associated indicators.

Problem criteria	Indicator(s)
Longstanding	Has been a problem for a long time. Some see the problem as the ‘norm,’ and may have labeled it ‘unsolvable.’
Failed solutions	Several (often similar) attempts to solve the problem have been tried.
Emotionally draining	The problem absorbs mental/physical resources. For example, continually playing on the mind of the client and/or frequently raised during team meetings/staff discussions.
Multiple-agency	The problem will be related to another person/group of people. There might be multiple explanations behind why the issue is perceived as it is, but the client feels responsible for the problem.

Stage 2 focused on the during session elements of problem-cleaning, the co-creation of a ‘solvable’ problem and intervention design (see Figure 1). Within the problem-cleaning phase techniques were used that ‘cleaned’ assumptions and generalizations from clients’ problem descriptions. This helped co-create a ‘solvable’ description of the problem between the client, primary practitioner and consultancy team by re-labeling the problem each time the client outlined an insight to their situation (i.e., an assumption was revealed). Navy’s case contextualized this process when the problem was re-labeled from a *“fundamental cultural problem throughout the whole organization”* through several iterations to one framed as a “*difficult conversation”* (see Table 2). The questions and techniques used by the primary practitioner to assist this process included: initial checks; exploring previous solutions; describing a preferred future; searching for exceptions; revealing constraints; and video descriptions (cf. O’Hanlon and Wilk, 1987; de Shazer et al., 2007). See Table 4 for the essential questions associated with these techniques. These questions were based on our analysis and interpretations from the data within Studies 1 and 2, and were supported by extant literature (e.g., Watzlawick et al., 1974; de Shazer, 1985). In the context of our single-session problem-solving framework, they were used iteratively throughout a session. However, future research practitioners may wish to explore the individual use of a particular questioning technique within their existing needs analysis and problem-solving practices.

**TABLE 4:** Essential questioning techniques during the problem-cleaning stage.

Technique	Questions/exercises	Purpose
Initial checks	How specifically is this a problem *for you*? What would happen if you decided to do nothing? If a fire alarm went off, and we had to end the session right now, what would you try to do to resolve this problem if you had to try something? What do you need to leave with at the end of the session?	Enabled sessions to be goal-directed and enhanced the client(s) motivation to think differently about solutions. Ensured the client(s) had ‘ownership’ of the problem.
Exploring previous solutions	Client writes down everything they have ever done or tried to resolve the problem. Client looks for commonalities within their previous solutions and what assumptions this reveals they may have been making. Client groups their previous solutions by what made the problem worse, made no difference, or made the situation slightly better. Client looks for commonalities within their previous solutions (i.e., worse, no difference, better).	Helped reveal assumptions client(s) make about their problem. Helped reveal any contextual differences between previous solutions that had, or had not been effective.
Describing a preferred future	If you went to bed tonight, and when you were asleep a miracle occurred, and this problem disappeared overnight without you knowing the miracle had happened, how would you know in the morning that this problem no longer existed? What would be the first small signs that this problem no longer existed? Client draws their situation as if there was no longer a problem. Challenge the client to distinguish between the ‘nice to have’ and ‘need to have’ elements of their preferred future.	Enabled client(s) to avoid thinking causally about how and why the situation was a problem. Shifted focus to how client(s) would know if the situation was no longer a problem (links to identifying exceptions).
Searching for exceptions	Can you think of any times when the problem did not happen? Can you think of a time when your current situation was not a problem? When/Where does your problem not happen? When have aspects of your preferred future happened before?	Helped the practitioner(s) identify resources. Enabled client(s) to challenge their perception that the problem was all-pervading, had always been present, and that they were completely ‘stuck.’
Revealing constraints	Compare the current situation to an exception. What is present (or not present) in the exception that is different from the current situation? If you had to re-create this problem in an alternate universe, what would definitely have to be present?	Directed the practitioner(s) and client(s) to factors preventing the issue from being resolved.
Video descriptions	Client describes their situation as if they were watching it on a television screen. Client draws their problem, preferred future, or exception.	Helped the practitioner(s) separate the facts of a situation from the meaning attached to it by client(s).

Once a ‘solvable’ problem frame had been negotiated, the EIS psychologists would gather unique situational or client factors (termed ‘resources’) useful for intervention design. For example, in Navy’s case, capturing information on the best location and time for the ‘difficult conversation’ to take place were resources gathered to assist with intervention. Resources were often gathered during the problem-cleaning stage (see Figure 1). The observation team assisted this process by ‘mapping’ the critical insights and resources revealed by the client during this stage. Where relevant, the team would integrate these resources into intervention design (cf. Miller, 2017).

Stage 3 of the problem-solving framework focused on the effectiveness of the session and intervention using various methods of post-session feedback. This primarily involved the use of a post-session evaluation form (cf. Campbell, 1999) and a social validation interview consistent with previous single-session therapy approaches (see Fry, 2012; Slive and Bobele, 2012). This allowed for an evaluation of the effectiveness of the single-session and whether any further support was required (e.g., taking a similar or different approach).

## Overall Discussion

This research summarized an in depth, 6-year long study of single-session problem-solving within a world leading provider of sports science and sports medicine services in the United Kingdom. The overall broad aim of our research was to generate an applied framework of single-session problems solving for use within an elite sport context. Two separate, but interrelated studies helped address this aim and the systematic approach used helped generate the framework outlined in Figure 1. This framework goes beyond any previous single-session problem-solving research in sport, and beyond such research in other contexts that have only explored the application of a *single* therapeutic model (e.g., Young et al., 2008). This is a new and meaningful step in revealing how several different brief therapeutic techniques can be *integrated* into one effective framework of single-session problem-solving. The multi-study approach, and depth provided on *how* the techniques and methods can be applied realizes research that stands alone regarding its complexity and value when exploring single-session problem-solving methods.

Our research provided a framework of problem-solving that ostensibly integrated techniques from several brief therapeutic approaches (e.g., brief, strategic, and solution-focused models of therapy). Although some question the philosophical and conceptual basis for such an integrative, eclectic approach to philosophy of practice, we, and others would counter with the suggestion that the techniques and approaches within our framework are clearly connected philosophically and conceptually (Eron and Lund, 1993; Geyerhofer and Komori, 2004; Poczwardowski et al., 2004). Specifically, brief, strategic, and solution-focused models of therapy were all influenced at a philosophical level by the ideas of Ludwig Wittgenstein (1953) and were impacted conceptually by the applied practice of Milton Erickson (1954, 1980) and theoretical perspectives of Gregory Bateson (1972, 1979). These shared influences have resulted in brief, strategic, and solution-focused models of therapy possessing a number of complimentary foundations (Eron and Lund, 1993; Geyerhofer and Komori, 2004). For example, the individual techniques integrated within our framework all shared an interactional, social-constructivist epistemology (Watzlawick et al., 1974; de Shazer, 1994). From this perspective, an individual’s experience of reality, the assumptions they make, and the meaning they give to their experiences are constructed through their interactions with other people, objects, events, and their language. The ‘negotiation’ or co-creation of problems to a solvable frame that was central to the single-session problem-solving approach described in our study was founded on an epistemological standpoint that aligned to this interactional, social-constructivist epistemology. So, despite drawing from multiple *individual* therapeutic approaches our framework has common philosophical and conceptual foundations. Further, and with particular reference to providing sport psychology services in an elite context, authors have recognized that some experienced practitioners use an eclectic underpinning paradigm that utilizes a variety of theoretical models to underpin their holistic service provision (see Friesen and Orlick, 2010).

A further conceptual implication related to the suggestion that the role of ‘language’ was a critical feature when applying our framework. Several steps and questioning techniques focused on how a problem was *described* by a client, and practitioners used a variety of problem-cleaning techniques to help the client redefine and re-describe a problem. This focus on language is consistent with some other brief therapies (e.g., solution-focused therapy, strategic therapy), where language was the *central* concept and mechanism for change. The language clients used to think about (i.e., internal dialogue), discuss and describe their problems contributed to both the maintenance of their problem and possible options for resolution. Aligned to the interactional, social constructivist standpoint, the single-session problem-solving framework outlined by our research is grounded in the notion that an individual’s reality is framed and limited by their use of language (see Wittgenstein, 1953). The use of problem-cleaning techniques that challenged a clients’ *description* (i.e., use of language) of their situation assisted them to reframe and co-create a ‘solvable’ frame of their problem. Once a solvable, behavioral description was formulated by the client, designing a suitable intervention was a relatively simple process as the new frame of the problem allowed the client to contemplate new possibilities and solutions.

A number of practical implications for a range of helping domains can be realized from our findings. The main implication is the framework for single-session problem-solving displayed in Figure 1. The framework, and the individual techniques within it represent a new and novel way of approaching problem-solving that is suitable for problems at an individual, team, or organizational level. The questioning techniques offered within the framework (see Figure 1 and Table 4) provide applied practitioners with a mechanism to work with clients to help them solve complex problems, that have been longstanding (i.e., ‘stuck’) via a reframing and problem-cleaning approach (i.e., co-creating a ‘solvable’ problem). The framework also offers practitioner groups within the high-performance sport system in the United Kingdom, and perhaps elsewhere, with a means of resolving problems that would traditionally be described as ‘cultural’, ‘organizational’ or ‘people-related’ problems, within a single-session, when other attempts to resolve the issue have failed (see Wagstaff, 2019; Sly et al., 2020). Given that single-session therapies and brief approaches have transcended across therapeutic domains, the complete framework, or some of the individual techniques outlined within it may have relevance and impact within other support settings outside of sport (e.g., social work, mental health services, occupational and organizational psychology).

Our findings, and the use of a single-session approach also have potential implications for how we as sport psychologists view therapeutic alliance and the client-practitioner relationship. Specifically, across sport and other counseling domains, therapeutic alliance (i.e., the client-psychologist relationship) has been evidenced as an essential component of intervention effectiveness in talking therapy (e.g., Baldwin et al., 2007; Andersen and Speed, 2010; Mack et al., 2019). Our framework, nor SST as a broad practice philosophy allows time for a traditional therapeutic alliance to develop. In a family therapy context, some practitioners have noted how their beliefs and values on the sufficiency of therapeutic alliance led them to question adopting a single-session approach (see Fry, 2012). We did not find evidence for such assertions within our data; but, such questions were not a focal point of our research. Future research in a sporting context should look to more explicitly explore how adopting briefer, single-session approaches may impact on a practitioner’s beliefs and values and their applied practice philosophy. Such research could focus on issues related to therapeutic-alliance and on the barriers, challenges and doubts practitioners may experience when shifting their practice philosophies to align with briefer approaches (see Fry, 2012; Pitt et al., 2015b).

Despite the strength of our work, there are some limitations to the reach and impact of our findings. Specifically, our framework was developed within a Westernized high performance sport culture. As such, although the design and methods used within our work would not lead us to generalize, there are some clear cultural limitations to where the framework and approach may be effective. Future research could look to explore the effectiveness of the approach across a range of cultures and across a range of competitive levels and settings. Although the case-study design adopted in Study 2 provided some indication of the effectiveness of the framework, we would suggest further testing is required to provide more rigorous evidence (see Hymmen et al., 2013). Further, we have not provided evidence for the efficacy of our framework. Although some would argue that controlled experimental studies are not suitable in a range of therapeutic settings, and designs that demonstrate effectiveness are deemed a suitably valuable evaluation of practice, efficacy studies are considered the ‘gold standard’ for measuring if a treatment works (e.g., Seligman, 1995; Anderson et al., 2002). We would suggest future researchers should seek to both broaden the contexts and cultures within which the framework could be deemed as effective, and perhaps try to design studies that enable efficacy to be tested. A final set of limitations relate to the operationalization of the broad approach. First, the practice context within the EIS allowed a team model of practice to be developed; and although we would suggest that the consultancy team is not a prerequisite for using the problem-solving framework, we found that they play an important role in supporting the primary practitioner. It is important to note that not all sport psychologists have access and opportunity to work within such teams (see Pitt et al., 2015a). Related to this, future research is needed to further understand the ethical (e.g., shared confidentiality), professional practice and philosophy and training needs when seeking to adopt a consultancy team support model. Second, although the framework aligned with other single-session therapies, and had close links to a range of brief therapies, there needs to be recognition that each single-session lasted an extended period of time (usually several hours). As such, the overall approach would perhaps not be best described as ‘brief,’ despite it being short when compared to more traditional change or problem-solving interventions within sport psychology (e.g., a cognitive-behavioral psychological skills program) applied over several sessions and/or weeks (see Barker et al., 2020). Finally, within our assessments of effectiveness, we concentrated on the short-term effectiveness of the problem-solving framework; future research should consider exploring whether this impact is maintained or has any long-term cultural benefits (cf., Sly et al., 2020).

In summary, our research, and new single-session problem-solving framework that we developed from it, offers practitioners across a range of psychology disciplines a potential way to solve problems within a single-session. Such frameworks are critical within sport psychology as our field moves quite rapidly beyond that of a discipline traditionally focused on developing athletes’ cognitive abilities to one that recognizes a broader range of therapeutic approaches and diverse client populations across a growing range of performance domains (Cruickshank and Collins, 2013; Sly et al., 2020). Furthermore, in contexts such as sport or counseling, where there are increased demands on time, accessibility and/or budget, frameworks of service delivery that are fast-impacting are particularly relevant and useful.

## Data Availability Statement

The datasets presented in this article are not readily available. Due of the potentially sensitive nature of the observations, interviews and case studies, the participants in the research did not grant us permission to share or let others read the full transcripts and/reflections from these data collections. However, they have granted permission for the quotes and case study detail that have been selected for inclusion in the article to be shared.

## Ethics Statement

The studies were reviewed and approved by Cardiff School of Sport Research Ethics Committee, Cardiff Metropolitan University (reference 13/02/04R). Participants provided their written informed consent to participate in this study.

## Author Contributions

TP contributed to research design, data collection, data analysis, and write up. OT and PL contributed to research design, data analysis, and write up. SH contributed to research design, and write up. MB contributed to research design. All authors contributed to the article and approved the submitted version.

## Conflict of Interest

PT, PL and MB are now employed by company Mindflick. The remaining authors declare that the research was conducted in the absence of any commercial or financial relationships that could be construed as a potential conflict of interest.
